# Implicit sequence learning in people with Parkinson’s disease

**DOI:** 10.3389/fnhum.2014.00563

**Published:** 2014-08-01

**Authors:** Katherine R. Gamble, Thomas J. Cummings Jr., Steven E. Lo, Pritha T. Ghosh, James H. Howard Jr., Darlene V. Howard

**Affiliations:** ^1^Cognitive Aging Lab, Department of Psychology, Georgetown UniversityWashington, DC, USA; ^2^Department of Psychiatry, MedStar Georgetown University HospitalWashington, DC, USA; ^3^Department of Neurology, MedStar Georgetown University HospitalWashington, DC, USA; ^4^Medical Faculty Associates, George Washington UniversityWashington, DC, USA; ^5^Cognitive Aging Lab, Department of Psychology, The Catholic University of AmericaWashington, DC, USA; ^6^Department of Neurology, Georgetown University Medical CenterWashington, DC, USA

**Keywords:** implicit learning, Parkinson’s disease, implicit sequence learning

## Abstract

Implicit sequence learning involves learning about dependencies in sequences of events without intent to learn or awareness of what has been learned. Sequence learning is related to striatal dopamine levels, striatal activation, and integrity of white matter connections. People with Parkinson’s disease (PD) have degeneration of dopamine-producing neurons, leading to dopamine deficiency and therefore striatal deficits, and they have difficulties with sequencing, including complex language comprehension and postural stability. Most research on implicit sequence learning in PD has used motor-based tasks. However, because PD presents with motor deficits, it is difficult to assess whether learning itself is impaired in these tasks. The present study used an implicit sequence learning task with a reduced motor component, the Triplets Learning Task (TLT). People with PD and age- and education-matched healthy older adults completed three sessions (each consisting of 10 blocks of 50 trials) of the TLT. Results revealed that the PD group was able to learn the sequence, however, when learning was examined using a Half Blocks analysis (Nemeth et al., 2013), which compared learning in the 1st 25/50 trials of all blocks to that in the 2nd 25/50 trials, the PD group showed significantly less learning than Controls in the 2nd Half Blocks, but not in the 1st. Nemeth et al. (2013) hypothesized that the 1st Half Blocks involve recall and reactivation of the sequence learned, thus reflecting hippocampal-dependent learning, while the 2nd Half Blocks involve proceduralized behavior of learned sequences, reflecting striatal-based learning. The present results suggest that the PD group had intact hippocampal-dependent implicit sequence learning, but impaired striatal-dependent learning. Thus, sequencing deficits in PD are likely due to striatal impairments, but other brain systems, such as the hippocampus, may be able to partially compensate for striatal decline to improve performance.

## Introduction

Implicit sequence learning is the learning of relationships between events that occur sequentially in time, and occurs without intent to learn or awareness of what has been learned (Reber, 1989). This type of learning allows us to be sensitive to regularities in our environment, and adapt to changes in physical and social cues. Implicit sequence learning contributes to many behaviors, including learning new languages (Kuhl, 2004), comprehending and producing complex sentences (Illes et al., 1988; Grossman et al., 2001), understanding and interpreting social cues (Lieberman, 2000), and walking, as well as various types of rehabilitation (Abbruzzese et al., 2009).

Striatal dopamine has been shown to be necessary not only for executing sequenced movements (Matsumoto et al., 1999), but also for motor sequence *learning* (Badgaiyen et al., 2007), and a genotype relating to dopamine availability is related to improved *implicit* sequence learning (Simon et al., 2011b). Neuroimaging studies support these findings regarding dopamine, showing that striatal activation is related to successful implicit sequence learning (Rieckmann et al., 2010; Dennis and Cabeza, 2011; Simon et al., 2012), and that white matter integrity of a striatal-dorsolateral prefrontal cortex tract mediates age differences in implicit sequence learning (Bennett et al., 2011).

Parkinson’s disease (PD) is a neurodegenerative disorder characterized by motor impairments (Hoehn and Yahr, 1967) that result from dopamine cell death in the substantia nigra (Fearnley and Lees, 1991), with a resulting depletion of dopamine within the striatum (Kish et al., 1988). These dopamine declines also play a role in more general gait disturbances, which leads to an increased risk of falls (c.f., Rodriguez-Oroz, 2012), such that up to 70% of people with PD report at least one fall a year (Bloem et al., 2003). Nonetheless, the extent to which underlying implicit sequence learning abilities are impaired or intact in people with PD is still not clear, with the literature yielding mixed results. The present paper aims to address two potential sources of such variation.

First, it is possible that when deficits in sequence learning are seen, they are due to deficits in motor sequencing, rather than in the underlying learning itself. This is because much of what is known about sequence learning in PD has come from studies using the Serial Reaction Time (SRT) task (Nissen and Bullemer, 1987). In the SRT, participants respond to a series of stimuli presented in four locations across the computer screen, with these locations following a particular pattern or regularity. Sequence learning is typically assessed by removing the regularity at one or more points in training, to see whether response times are disrupted; the amount of disruption indicates the amount of sequence learning. Parkinson’s participants usually show impaired SRT learning compared to healthy older adults (Wilkinson et al., 2009; van Tilborg and Hulstijn, 2010; Schendan et al., 2013), but since the SRT has a large motor component, it is possible that motor deficits that occur in PD prevent this group from revealing learning. Additionally, because each event only occurs after a response to the previous event, participants’ responses control the timing between events, and these interstimulus intervals have been shown to affect the extent to which learning occurs and/or is expressed (Howard et al., 2007). Therefore, it is possible that different motor abilities in PD participants affect their ability to learn, or to express learning, in the SRT. A verbal version of the SRT attempts to overcome some of the potential effects of motor involvement, but results in PD groups are mixed in these studies (Smith et al., 2001; Smith and McDowall, 2004, 2011). Because the verbal SRT also requires a response to each event, a verbal response sequence must be produced rapidly in order to reveal learning. This requirement for rapid verbal responding might put PD participants at a disadvantage compared to healthy controls because they often have speech difficulties (Forrest et al., 1989; Sapir et al., 2008; Plowman-Prine et al., 2009).

The second potential source of variation across studies is that the way in which learning is measured in the SRT makes it difficult to determine the differential contributions of the underlying brain regions. Although the striatum is involved in implicit sequence learning in healthy participants, there is also hippocampal involvement (Rieckmann et al., 2010; Dennis and Cabeza, 2011), and the relative importance of these different learning systems varies across training and across different subject populations. Because in the typical SRT task, sequence learning is measured at only one, or a few points in training, it cannot distinguish hippocampal from striatal contributions to learning.

In the present study, we aimed to address both of these issues by using the Triplets Learning Task (TLT), which was derived from the SRT, but examines implicit sequence learning with no motor sequencing component (Howard et al., 2008). In the version of the TLT used here, on each trial participants view two red cues and respond to the location of a third cue, a green target, whose location is probabilistically predicted by the location of the first red cue. Perceptual sequence learning occurs as participants learn the predictive relationship between the first cue and the target while only responding to the target. Thus, there is no motor sequence to be learned, and a minimal motor response involved. Furthermore, in the present study, 80% of the time, the position of the first event in a triplet predicted the location of the third event (High probability trials), whereas on 20% of the trials it did not (Low probability). The amount of sequence learning is assessed by comparing performance on High and Low probability triplets throughout training. During TLT training, participants completed 30 blocks of trials, with brief breaks between each block.

A recent paper suggested that the relative importance of the striatal and hippocampal learning systems varies *within* blocks (Nemeth et al., 2013). Nemeth et al. compared sequence learning between healthy older adults and those with Mild Cognitive Impairment (MCI), a condition associated with declines in medial temporal lobe structure and function, but intact striatal function. Overall analyses revealed a learning difference between the healthy and MCI groups, with the MCI group showing no learning. However, when learning was examined separately in the 1st Half Blocks (first 40 of 80 trials in each block) compared to the 2nd Half Blocks (second 40 of 80 trials in each block), a different pattern of group differences emerged. In the 1st Half Blocks, the MCI group again showed no learning, and therefore less than the Controls, while in the 2nd Half Blocks, the MCI group showed learning equal to the controls. Nemeth et al. (2013) hypothesized that the 1st Half Blocks involved recall and reactivation of the sequence learned, thus reflecting learning dependent on the medial temporal lobe/hippocampus, while learning in the 2nd Half Blocks involved more automated and proceduralized behavior of learned sequences, and thus reflected striatal-based learning. This hypothesis is consistent with a processing-based model, which suggests that the hippocampus is involved in repeated encoding and reconsolidation, but may disengage as proceduralization occurs (Henke, 2010). The present study provided an opportunity to test this hypothesis, because Nemeth et al.’s (2013) interpretation would predict a double dissociation, such that, in contrast to Nemeth et al.’s MCI participants who showed impaired learning only in the 1st Half Blocks, our PD group should be impaired only in the 2nd Half Blocks when learning reflects striatal involvement.

In this study, we used the TLT to examine implicit sequence learning in people with PD compared to an age- and education-matched healthy older adult Control group. We predicted that people with PD would show less learning in the TLT than the Control group, but that this difference would be driven by the PD group showing less learning in the 2nd Half Blocks compared to Controls, but not in the 1st Half Blocks.

## Materials and methods

### Participants

Participants included 27 people with PD (*M* Age = 64.59, *SD* = 5.75, 10 females) and 30 age- and education-matched healthy older adult Controls from the Washington, DC community (*M* Age = 66.47, *SD* = 5.32, 20 females). Additional group comparisons can be found in Table 1. All participants in the Parkinson’s group had been diagnosed by a neurologist, and were referred to the lab from MedStar Georgetown University Hospital. PD participants were in Hoehn and Yahr (1967) stages 1 to 2.5 (Median = 1) and were taking their usual anti-Parkinsonian medication at the time of test. Fourteen participants (four PD and ten Controls) were removed using various *a priori* cutoffs, and were not included in participant characteristics or analyses presented here^1^.

**Table 1:** **Parkinson’s disease and healthy control participant characteristics**.

	**Parkinson’s disease participants**	**Control participants**	***t***	***p***
**Years of Education**	18.11 (2.75)	16.83 (3.52)	−1.51	0.136
**Self-reported Overall health (out of 5)**	3.93 (0.82)	4.27 (0.87)	1.45	0.153
**Geriatric Depression Score**	2.00 (2.15)	0.90 (1.56)	−2.23	0.030
**Montreal Cognitive Assessment**	27.11 (1.72)	27.97 (1.50)	2.01	0.050
**SCOPA-COG overall score**	31.41 (2.85)	n/a
**WAIS-III Digit Span Forward**	10.70 (2.20)	10.23 (2.03)	−0.84	0.405
**WAIS-III Digit Span Backward**	7.00 (1.88)	8.36 (2.06)	2.49	0.016
NAART-35*	9.70 (5.50)	8.23 (5.71)	−0.99	0.328

Standard deviation in parentheses. *In the NAART-35, a lower score is indicative of higher verbal intelligence.

All participants received monetary compensation for their participation, and all methods were approved by the Georgetown University Institutional Review Board.

### Tasks

#### Triplets learning task

**Figure 1:**
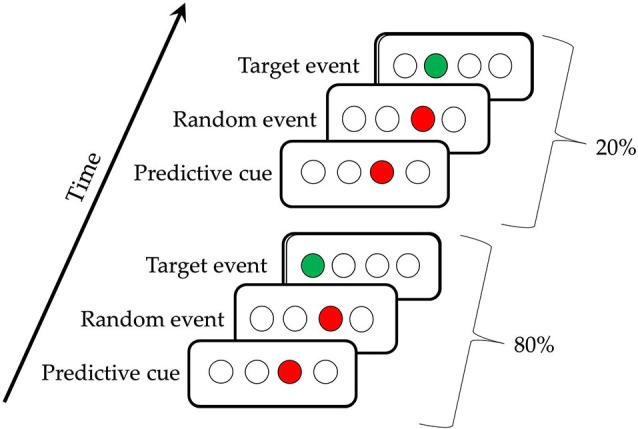
**The Triplets Learning Task**. The first triplet indicates a High Probability triplet (e.g., 3r1) and the second triplet indicates a Low Probability triplet (e.g., 3r2), for a participant receiving the regularity 1r2r4r3r.

The TLT is illustrated in Figure 1; participants saw four evenly spaced open circles (0.5° each, on a screen that was 12° at 56 cm viewing distance) in the middle of a computer screen (Howard et al., 2008). Each trial consisted of three successively presented events, two cues and a target, which together made a *triplet*. The first cue consisted of one of the four circles filling in red for 120 ms, followed by a 150 ms interstimulus interval, then a second cue (the same or a different circle) filling in red for 120 ms, another 150 ms interstimulus interval, and then a target consisting of a circle filling in green. This green target remained lit until the participant responded correctly. Participants responded to targets by using their left and right middle and index fingers to press keys, “z,” “x,” “.” and “/” on a standard keyboard in response to the first, second, third, and fourth circles, respectively. If using two hands was uncomfortable, PD participants were offered the opportunity to use a stimulus-response box with their hand of choice, with four keys across corresponding to the four circles. Twenty-three PD participants used the keyboard, and four used the S-R box. There were 50 trials per block, 10 blocks per session, and three sessions for each participant, all completed in a single visit to the laboratory. End of block accuracy and reaction time (RT) feedback was given after every block to direct participants to achieve 92% accuracy, with the aim of matching the groups on overall accuracy. If their accuracy was ≥ 94%, participants were shown the instruction, “Focus more on speed,” and if it was ≤ 90%, they saw a message to, “Focus more on accuracy,” otherwise, participants were told to, “Please continue,” suggesting that they were performing optimally. Participants took an average break of 90 s between blocks (range: 35–119 s).

The triplet sequence contained a second-order regularity, in that the location of the first red cue predicted the location of the target (the green event), with the location of the second red cue being random (Howard et al., 2008). Referring to the circles as 1, 2, 3 and 4 from left to right, one possible pattern is 1r2r3r4r, where r is one of the four circles chosen randomly. Participants receiving this pattern would see the High frequency triplets 1r2, 2r3, 3r4, and 4r1 on 80% of the trials, and the Low frequency triplets (e.g., 1r3, 2r4) on the remaining 20% of trials. This 80:20 proportion was chosen because it has been shown to yield learning in healthy older adults with the amount of training used here (Howard et al., 2008; Simon et al., 2011a). There were six unique triplet patterns counterbalanced across participants (1r2r3r4r, 1r2r4r3r, 1r3r2r4r, 1r3r4r2r, 1r4r2r3r, and 1r4r3r2r), so that a given triplet (e.g., 1r4) was High frequency throughout training for some participants, but Low for others. With four possible positions and three circles being lit per trial, there are 64 possible triplets. Thus, for each pattern, there were 16 possible High frequency triplets, and 48 possible Low frequency triplets.

#### TLT recognition test

In the TLT Recognition test following the three sessions of the TLT, participants were shown each of the 64 triplets on the computer screen, presented with the same timing as during the TLT. Participants were asked to observe each triplet without responding to the target, and then to report via a keypress if they thought each individual triplet occurred “more often” or “less often” during training.

### Procedure

Participants first provided Informed Consent. They then completed Health screening and Biographical questionnaires, as well as the short form of the Geriatric Depression Scale (Brink et al., 1982). Next, they were read instructions for and completed three sessions of the TLT. They were told that they would see four open circles, two of which would first fill in red, followed by a third that would fill in green, and that they were to respond to the location of the green target using the corresponding keypress. There were 10 blocks in a session, and participants were told they could take short breaks between blocks, but asked to not leave the room. They were given longer breaks between each session, where they could get up and walk around. Following the third session, participants’ explicit knowledge was tested in two ways: first, through the TLT Recognition test, and second, a verbal questionnaire probing strategies and their ability to reproduce the high frequency triplets.

Following the Triplets questionnaire, all participants completed a battery of tests, including the Montreal Cognitive Assessment (MoCA; Nasreddine et al., 2005), the Wechsler Adult Intelligence Scale-III Forward and Backward Digit Span (Wechsler, 1997), and the North American Adult Reading Test-35 (NAART-35; Uttl, 2002). For the PD participants, the battery also included the Scales for Outcomes of PD – Cognition (SCOPA-COG; Marinus et al., 2003). Control participants were not tested on the SCOPA-COG, as it is a test of cognition specific to people with PD. Finally, participants were debriefed and compensated. The entire day of testing lasted approximately two and a half hours.

### Statistical analysis

In the following, we first examined the implicitness of learning via the Recognition test. For each person, we calculated the proportion of High probability and Low probability triplets that were rated as having “occurred more often.” We then subjected these to a mixed-design 2 (Group) × 2 (Triplet type) ANOVA, with Group (PD vs. Control) as a between-subjects and Triplet type (High vs. Low probability) as a within-subjects factor.

We then examined overall sequence learning in the TLT task, first using accuracy and then RT. For accuracy, for each person we determined the proportion of correct responses on the TLT separately for High and Low probability triplets, and then subjected these data to a mixed-design 2 (Group) × 2 (Triplet type) ANOVA, again, with Group varying within-subjects and Triplet type between-subjects. For RT, we calculated median RTs separately for High and Low probability triplets in each block of 50 trials to minimize the effects of outliers. We then averaged the block medians to obtain the mean of median RT across all 30 blocks of training for each person, and subjected these data to a mixed-design 2 (Group) × 2 (Triplet type) ANOVA, with Group as a between-subjects and Triplet type as a within-subjects factor.

Our final set of analyses examined learning broken down by the 1st vs. 2nd Half Blocks. To do this, we first combined the 1st Half of all Blocks (first 25 of 50 trials per block) and the 2nd Half of all Blocks (second 25 of 50 trials per block) across all 30 training blocks. We then calculated the mean of median RT, and ran a mixed-design 2 (Group) × 2 (Half Blocks) × 2 (Triplet type) ANOVA, with Group varying between-subjects and Half Blocks and Triplet type within-subjects. Finally, to examine how this Half Block effect evolved with training, we divided the 30 blocks into six epochs, each containing five blocks. For each person we calculated the mean of median RT for High and Low probability triplets for each Half Block of each epoch, and used these means to calculate the RT Triplet type effect (RT to Low – RT to High probability triplets). We then submitted these Triplet type effects to a mixed-design 2 (Group) × 2 (Half Blocks) × 6 (Epoch) ANOVA, with Group varying between-subjects and Half Block and Epoch within-subjects.

All analyses were run using a significance level of α = 0.05, with Bonferroni correction used for all follow-up *t*-tests.

## Results

### TLT recognition test

**Figure 2:**
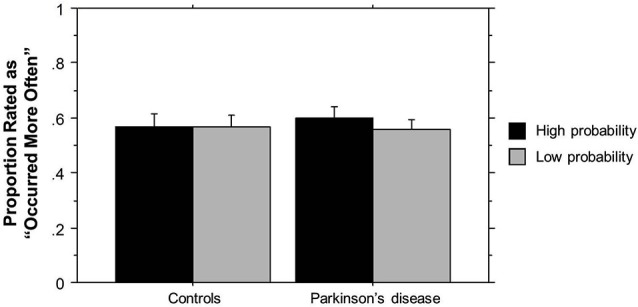
**TLT Recognition task rating of the frequency of triplets’ occurrence by Group**. Bars indicate standard error.

The ANOVA on ratings of triplet frequency yielded no significant effects (*p*’s > 0.10). As can be seen in Figure 2, participants did not rate High probability triplets as having occurred more frequently than Low probability triplets, and there was no difference in ratings between the two groups. These results suggest that participants were not aware of the triplet frequencies seen during training, and thus any learning that occurred was largely implicit. The post-Triplets verbal questionnaires also gave no indication that participants had awareness of the pattern they had encountered.

### TLT accuracy

The ANOVA on accuracy in the TLT yielded a significant main effect of Triplet type (*F*_(1,55)_ = 4.90, *p* = 0.031), such that accuracy was higher for High (*M* = 0.965, *SD* = 0.023) than for Low probability triplets (*M* = 0.0960, *SD* = 0.029). There was no significant main effect of or interaction with Group, *p*’s > 0.10. The fact that the groups did not differ significantly in overall accuracy allowed us to analyze RT without being concerned about the groups differing in a speed-accuracy tradeoff.

### TLT reaction time

**Figure 3:**
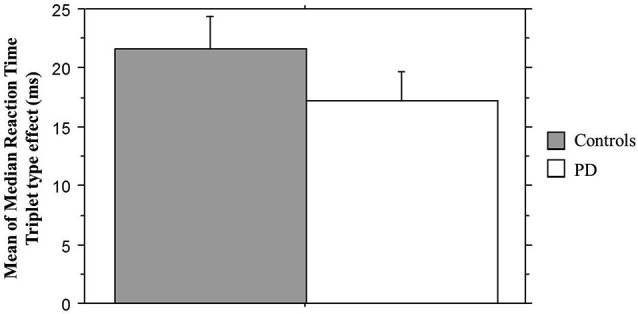
**Mean of median reaction time Triplet type effect (RT to Low probability – RT to High probability triplets) by Group**. Bars indicate standard error.

We next examined sequence learning using RTs; Triplet type effects (RT to Low – RT to High probability triplets), which provide our measure of sequence learning, are displayed in Figure 3. An ANOVA on mean of median RTs yielded a significant main effect of Triplet type (*F*_(1, 55)_ = 112.42, *p* < 0.001), such that RT was faster to High than Low probability triplets, signaling that sequence learning had occurred. Neither the main effect of Group (*p* > 0.08) nor the Group × Triplet type interaction (*p* > 0.10) was significant. Follow-up paired *t*-tests comparing response time to High and Low probability triplets within each Group indicated that both groups responded significantly faster to High than to Low probability triplets (Table 2). Thus, both Groups showed sensitivity to triplet probabilities, and they did not differ significantly from each other in the amount of sequence learning as assessed by the Triplet type effect, even though as Figure 3 indicates, the PD group was in the direction of showing less learning than the Control group.

**Table 2:** **Mean of median reaction time for each Triplet type by Group**.

	**High probability triplets**	**Low probability triplets**	***t***	***p***
**Parkinson’s disease**	532.32 (79.91)	549.53 (80.12)	−6.98	< 0.001
**Healthy Controls**	494.39 (84.52)	516.04 (84.14)	−8.09	< 0.001

*Standard deviation in parentheses*.

### TLT reaction time: half blocks comparison

If, as Nemeth et al. (2013) propose, the 1st Half Blocks primarily reflect hippocampal-based learning, and the 2nd Half striatal-based learning, then the above whole block analysis could be hiding important group differences in our study. Their MCI patients, a group that has medial temporal lobe deficits, revealed impaired learning compared to healthy controls in the 1st Half Blocks, but not in the 2nd Half Blocks. We reasoned that if Nemeth et al.’s interpretation was correct, then we should see the opposite pattern. That is, given their striatal deficits, Parkinson’s participants should show less learning than Controls in the 2nd Half Blocks, but not in the 1st.

**Figure 4:**
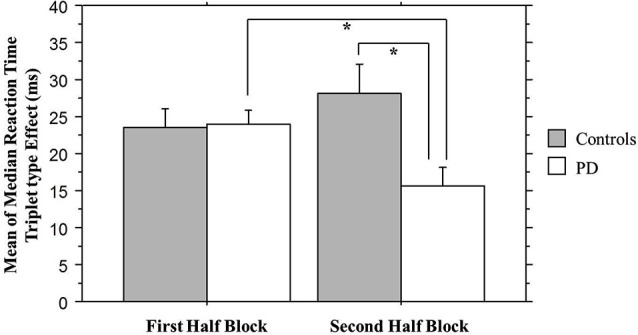
**Overall Half Block Triplet type effect (RT to Low Probability triplets – RT to High Probability triplets) by Group**. Asterisks indicate a significant difference, *p*’s = .01. Bars indicate standard error.

We combined the 1st Half of all Blocks (first 25 of 50 trials per block) and the 2nd Half of all Blocks (second 25 of 50 trials per block) across all 30 training blocks; the Triplet type effects from this analysis can be seen in Figure 4. The ANOVA on mean of median RTs yielded significant main effects of Half Blocks (*F*_(1,55)_ = 41.68, *p* < 0.001) and Triplet type (*F*_(1,55)_ = 178.17, *p* < 0.001). Neither the main effect of Group (*p* > 0.10), nor the interaction of Group × Triplet type was significant (*p* > 0.08). However, there was a significant interaction of Group × Half Blocks (*F*_(1,55)_ = 5.07, *p* = 0.028), and most important, a significant three-way interaction of Group × Half Blocks × Triplet type (*F*_(1,55)_ = 7.60, *p* = 0.007). Follow-up analyses using Bonferroni correction revealed that, as predicted, the Triplet type effect was significantly greater for the Control than the PD group in the 2nd Half Blocks (*t*_(55)_ = 2.56, *p* = 0.013), but not in the 1st (*t*_(55)_ = 0.924, *p* > 0.10). Additionally, the PD group showed significantly less learning in the 2nd than the 1st Half Blocks (*t*_(26)_ = 2.66, *p* = 0.013), but the Control group did not.

**Figure 5:**
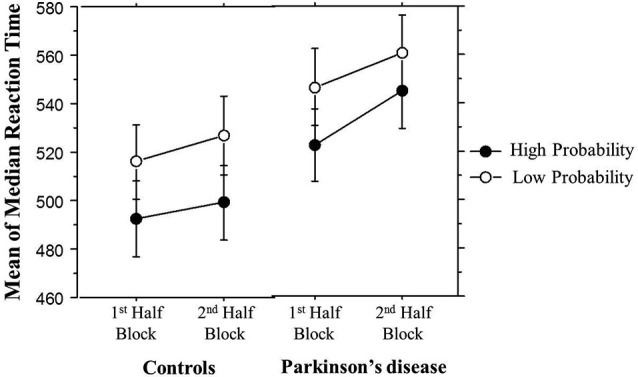
**Overall mean of median reaction time (ms) by Half Block and Group**. Bars indicate standard error.

It is possible that learning differences in the 2nd Half Blocks were due to fatigue building up within a block in the PD but not the Control group. In order to examine this possibility, Figure 5 breaks down the Triplet type effects displayed in Figure 4 by showing the response times to High and Low probability Triplet types separately by Group and Half Blocks. The significant Group × Half Blocks interaction reported above indicates that, as can be seen in Figure 5, overall, PD participants did slow down more than Controls from the 1st to the 2nd Half Blocks, consistent with a fatigue interpretation. However, Figure 5 also makes clear that (consistent with the significant Group × Half Blocks × Triplet type interaction), this Group difference is largely driven by the High probability Triplet type. Difference scores were calculated to measure the change in response time from the 1st to 2nd Half Blocks in High and Low probability triplets separately, and Bonferroni-adjusted unpaired *t*-tests examined if the Control and PD groups differed in this RT change for either Triplet type. This revealed a significant difference between the two groups for High probability triplets, such that the PD group showed a greater RT increase from the 1st to 2nd Half Blocks than Controls, but there was no group difference in the RT change for Low probability triplets (Table 3). Thus, the Group difference seen in learning in the 2nd Half Blocks is not likely due to an overall fatigue effect in the PD group, as both groups showed slowing from the 1st to the 2nd Half Blocks, and the PD group showed more slowing than Controls only on the High probability triplets.

**Table 3:** **Mean reaction time increase from 1st to 2nd Half Blocks by Triplet type and Group**.

	**Parkinson’s disease**	**Healthy Controls**	***t***	***p***
**High probability triplets**	22.46 (19.98)	6.61 (15.44)	−3.37	0.001
**Low probability triplets**	14.20 (18.22)	11.09 (18.70)	−0.06	0.528

*Standard deviation in parentheses*.

**Figure 6:**
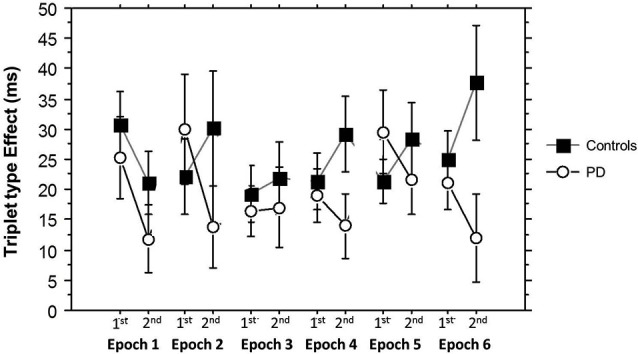
**Half Block Triplet type effect (RT to Low Probability triplets – RT to High Probability triplets) by Group and Epoch**. Bars indicate standard error.

Finally, Figure 6 shows how the Triplet type effect evolved across training. An ANOVA on these Triplet type effects yielded a significant main effect of Group (*F*_(1,53)_ = 4.71, *p* = 0.03) and a significant interaction between Group and Half Block (*F*_(1,53)_ = 8.60, *p* = 0.005), but no significant interactions between Half Block and Epoch. In the PD group, learning was consistently higher in the 2nd Half Blocks than the 1st, while in the Control group, epoch 1 showed the same pattern as the PD group, but in the remainder of training, learning was higher in the 1st than the 2nd Half Blocks. This is consistent with the conclusion that the Half Block difference in Triplet type effects across groups is present throughout most of training, with learning being more hippocampal-based in the PD group, and more striatal-based in the Control group. The sizes of these triplet type effects are consistent with those shown in previous studies using the TLT (Simon et al., 2011b, 2012).

## Discussion

In the present study, we found that people with PD were able to acquire implicit, perceptual, higher-order sequences in the TLT. When we examined overall learning, the PD group did not differ significantly from the Controls, but when analyzing learning through the Half Blocks analysis (Nemeth et al., 2013), we found that the PD group showed less learning than the Controls only in the 2nd Half Blocks, where performance is hypothesized to reflect striatal-based learning. This provides a double-dissociation with the findings of Nemeth et al., in that their MCI participants showed the reverse pattern, revealing less learning than the Controls only during the 1st Half Blocks which are thought to reflect hippocampal-dependent learning.

Our results are unlikely due to group differences in explicit, or declarative knowledge about the sequence in either group, as post-training measures showed that learning was implicit. Our results are also unlikely to be due to effects of motor sequencing or motor deficits in the PD group, as there is no motor sequencing in the TLT, and a highly reduced motor component for responding. Additionally, the learning difference seen in the 2nd Half Blocks does not seem to be due to a fatigue effect in the PD group, as their response time only slowed more than Controls to High probability triplets. Thus, we suggest that our results reflect a striatal-based sequence learning deficit in people with PD that can be tapped through examining learning in the 2nd Half Blocks in the TLT.

The hypothesis of the hippocampus being involved in recall and reconsolidation of previously learned sequences, and the striatum involved in proceduralization of that knowledge (Nemeth et al., 2013), is consistent with a processing-based model (Henke, 2010). This model suggests that the hippocampus is important for repeated encoding and reconsolidation, but the hippocampus may then disengage as information becomes proceduralized (Henke, 2010), which Nemeth et al. would suggest is when the striatal system largely underlies learning. Thus, the results from the Nemeth study and our own indicate that the Half Blocks analysis may be more analytic in characterizing learning deficits in clinical populations, in that it can help to reveal deficits in specific learning systems.

Performance of clinical populations in tasks that are hypothesized to differentially rely on the striatum or the hippocampus can help to parse out the involvement of each of these systems in different aspects of a task. The Nemeth et al. (2013) study showed this with a MCI group, and the present study with a PD group, but other studies have shown a similar dissociation. Two recent studies examined feedback-based learning, a type of learning typically shown to be impaired in PD (Foerde and Shohamy, 2011; Foerde et al., 2013). These studies tested PD and amnesiac participants, and found a double-dissociation, in that *immediate* feedback-based learning was impaired in PD but not amnesia, suggesting that it relies on the striatum. In contrast, when *delayed* feedback was given on some trials, learning was impaired in amnesiacs, but intact in people with PD, suggesting that this type of learning relies on the hippocampus. Similar to results in the present study, Foerde and Shohamy (2011) and Foerde et al. (2013) showed that clinical groups can help reveal how different systems subserve different components of learning in the same task. These examples also make clear that asking whether learning is impaired in a given group for a given task is phrasing the question too broadly, because most learning tasks call upon more than one neural system.

Our results must be interpreted in light of several limitations. First, all of our PD participants were on their typical medication at the time of test, because we wanted to examine how they learn and perform in the state in which they function on a daily basis. Additionally, we used higher-order probabilistic sequences, which again, was to model the types of associations with which people come into contact frequently. Our findings are similar to other studies examining sequence learning in people with PD that have shown impaired learning compared to controls (Wilkinson et al., 2009; van Tilborg and Hulstijn, 2010; Schendan et al., 2013), but in previous work, sequences were often deterministic (Ferraro et al., 1993), or participants were tested off of medication (Seidler et al., 2007), so the present study adds to the literature by testing a different type of learning in medicated participants. Nonetheless, our decision to test PD participants while they were medicated limits our ability to determine whether our results are due to medication or the disease itself. Second, we do not have measures of PD participants’ compliance to their medication, nor of where they were in their cycle of medication at the time they were tested. This is important because some participants may have been tested at the peak of their medication dose, when striatal dopamine levels should be high, while others may have been tested close to the time of their next dose, when striatal dopamine levels may have been lower. Future studies should examine both of these aspects of anti-Parkinsonian medication. Third, because we interpret our results in terms of a double-dissociation with the results of Nemeth et al. (2013) with MCI participants, some caution is in order. The two clinical groups were not only tested in different studies, but also using different tasks. Nemeth et al. used the Alternating SRT task (Howard and Howard, 1997), which not only has a motor sequencing component, but also has 80-trial blocks compared to the 50-trial blocks used here. It is encouraging that their hypothesis concerning the neural bases of Half Blocks is supported by our results, even though we used a different sequence learning task. Nonetheless, this double-dissociation with MCI and PD participants should be examined in a single study using the same task.

In conclusion, the present study examined whether people with PD could acquire implicit sequences in their environment while on their standard, daily medication. Results indicated that when on anti-Parkinsonian medication, people with PD are able to acquire implicit probabilistic sequences in a task that has minimal motor involvement. However, compared to age- and education-matched controls, their learning was impaired in portions of the task hypothesized to reflect striatal-dependent learning, but not in portions of the task hypothesized to reflect hippocampal-based learning. Thus, we suggest that striatal-based components of sequence learning are impaired in people with PD, but that learning relying on other brain systems, such as the hippocampus which is relatively intact, may help to moderate overall performance and learning in people with PD.

## Conflict of interest statement

The authors declare that the research was conducted in the absence of any commercial or financial relationships that could be construed as a potential conflict of interest.

## Footnotes

^1^Three PD participants and five Controls scored below the cutoff for the Montreal Cognitive Assessment (<24), two Controls had low verbal intelligence scores (NAART-35, a score well below that of the normed values for people of a particular age and education level), one Control had a history of memory loss, one Control was prescribed but not taking anti-psychotic medication, and one PD participant had an average RT more than 2.5 time greater than the group average. The pattern of results remained the same when these participants were included in analyses.
